# Hunger and associated harms among injection drug users in an urban Canadian setting

**DOI:** 10.1186/1747-597X-5-20

**Published:** 2010-08-26

**Authors:** Aranka Anema, Evan Wood, Sheri D Weiser, Jiezhi Qi, Julio SG Montaner, Thomas Kerr

**Affiliations:** 1 British Columbia Centre for Excellence in HIV/AIDS, St. Paul's Hospital, Vancouver, BC, Canada; 2 Department of Medicine, Faculty of Medicine, University of British Columbia, Vancouver, BC, Canada; 3 Center for AIDS Prevention Studies, University of California San Francisco (UCSF), San Francisco, CA, USA; 4 Positive Health Program, San Francisco General Hospital, University of California San Francisco (UCSF), San Francisco, CA, USA

## Abstract

**Background:**

Food insufficiency is often associated with health risks and adverse outcomes among marginalized populations. However, little is known about correlates of food insufficiency among injection drug users (IDU).

**Methods:**

We conducted a cross-sectional study to examine the prevalence and correlates of self-reported hunger in a large cohort of IDU in Vancouver, Canada. Food insufficiency was defined as reporting "I am hungry, but don't eat because I can't afford enough food". Logistic regression was used to determine independent socio-demographic and drug-use characteristics associated with food insufficiency.

**Results:**

Among 1,053 participants, 681 (64.7%) reported being hungry and unable to afford enough food. Self-reported hunger was independently associated with: unstable housing (adjusted odds ratio [AOR]: 1.68, 95% confidence interval [CI]: 1.20 - 2.36, spending ≥ $50/day on drugs (AOR: 1.43, 95% CI: 1.06 - 1.91), and symptoms of depression (AOR: 3.32, 95% CI: 2.45 - 4.48).

**Conclusion:**

These findings suggest that IDU in this setting would likely benefit from interventions that work to improve access to food and social support services, including addiction treatment programs which may reduce the adverse effect of ongoing drug use on hunger.

## Background

There were between 155 and 250 million illicit drug users worldwide in 2009 [1], including an estimated 16 million injection drug users (IDU) [2]. IDU face multiple structural and behavioral barriers to accessing health care and social support services, which collectively serve to compound health risks and exacerbate poor health outcomes [3]. IDU are known to be vulnerable to developing nutritional deficiencies, and often simultaneously experience numerous forms of micro and macronutrient deficiencies [4,5].

Insufficient consumption of food among IDU has been associated with an array of harms. Caloric insufficiency has been correlated with decreased immune function [6] and elevated risk of receiving a positive tuberculin test [7]. Insufficient caloric intake has been additionally associated with increased risk of various health complications including invasive candidiasis, viral hepatitis, bacterial pneumonia, and various infections including subcutaneous and perianal abscesses among IDU [5]. Studies evaluating the prevalence of nutritional deficiencies among IDU have tended to focus on HIV-infected populations [8,9]. In high resource settings, self-reported hunger, a marker of food insufficiency, has been found to be particularly elevated among IDU living with HIV/AIDS [10]. Self-reported hunger has been additionally correlated with unprotected sex and risk of HIV transmission, which is believed to be a result of survival sex-trade involvement [11].

Little is known about the social and behavioral risk factors, independent of HIV infection, that contribute to food insufficiency among IDU. We therefore sought to evaluate the prevalence of and socio-demographic and drug-using characteristics associated with self-reported hunger in a cohort of HIV-negative IDU in British Columbia, Canada.

## Methods

The Vancouver Injection Drug Users Study (VIDUS) was initiated in May 1996. VIDUS has been described in detail previously [12]. Briefly, participants were eligible for inclusion in VIDUS if they had injected illicit drugs at least once in the past month, lived in the Greater Vancouver region and if they provided written informed consent. At baseline, and semiannually thereafter, VIDUS participants provide blood samples for laboratory analysis, and completed an interviewer-administered questionnaire. The VIDUS questionnaire elicits information about socio-demographic status, injection and non-injection drug use, HIV risk behavior, income generation, encounters with police, and health service utilization. The questionnaire is based on a previous instrument developed for adult drug users, and has been found to reliably identify factors associated with HIV infection and other harms [13]. Participants receive an honorarium of $20 CDN for each study visit. Ethical approval for VIDUS is obtained on an annual basis from the Providence Health Care/University of British Columbia Research Ethics Board.

Questions regarding hunger were first included in the VIDUS instrument in December 2005. All individuals who completed a baseline interview between December, 2005 and March, 2009 were included in this analysis. Hunger is considered to be a severe manifestation of food insecurity [14]. Self-reported hunger, the primary dependent variable, was defined as answering 'yes' to the question: "I am hungry, but don't eat because I can't afford enough food". This definition of current self-reported was extracted from a validated food insecurity scale published by Radimer/Cornell [14]. Independent from the validated scale, this individual statement has been tested, and shown to have good specificity and sensitivity when compared to dietary proxies of food insufficiency in five North American settings [15].

In this cross-sectional analysis, we examined the point prevalence of self-reported hunger among IDU in relation to the following socio-demographic variables: median age, gender (male vs. female), ethnicity (Aboriginal vs. other), incarceration in the last 6 months (yes vs. no). We also explored several markers of socioeconomic status including: a) downtown eastside residency (yes vs. no), which is a community known as Canada's poorest postal code, and is characterized by high prevalence of unstable housing and illicit drug use [16,17], b) unstable housing (single-room occupancy dwelling, shelter, hostel, treatment centre or no fixed address vs. apartment or house), and c) current education status (high school or greater vs. other). We also examined self-reported symptoms of depression in the past week (≥ 16 CES-D score vs. < 16 CES-D score). Illicit drug use behaviors considered included: a) daily heroin injection in the past six months (yes vs. no), b) daily non-injection crack/rock in the past six months (yes vs. no), c) daily injection cocaine in the past six months (yes vs. no), d) any injection or non-injection crystal methamphetamine in the past six months (yes vs. no), e) any injection or non-injection drug binge in the past six months, defined as '[a time when you] injected drugs more than usual, or used any non-injection drugs more than usual' (yes vs. no), f) daily alcohol use (≥ 4 drinks vs. < 4 drinks), and g) difficulty accessing drug/alcohol treatment (yes vs. no), and h) money spent on drugs per day (≥ CAD$50 vs. < CAD$50). All behavioral variable definitions were identical to previous reports [18].

Univariate statistics were used to determine factors associated with self-reported hunger. Categorical explanatory variables were analyzed using Pearson's Chi-Square test, and continuous variables were analyzed using the Wald test. Degrees of freedom (df) were equal to one (df = 1) for all variables, with the exception of the 'symptoms of depression' variable, which was equal to 2 (df = 2) in both unadjusted and adjusted analyses. A multivariate model was then prepared using an *a priori *defined approach whereby variables that were associated with food insufficiency (*p *< 0.05) in univariate analyses were included in a fixed logistic regression model. Wald Chi-Squared tests were applied to all categorical and continuous variables. The concordance index was used to determine the final model fit [19], and the tolerance and variance inflation factor and condition index were used to assess multi-collinearity of the final model. All statistical analyses were performed using SAS software version 9.1 (SAS, Cary, NC). All reported p-values were two-sided.

## Results

A total of 1,053 participants were eligible for the present analyses. Overall, the median age was 41.6 years [interquartile range (IQR) 34.9 - 47.5]; 353 (33.5%) were female; and 307 (29.2%) self-identified as being of Aboriginal ancestry. Overall, 544 (51.7%) of participants had accessed a food bank or meal program in the past six months. A total of 681 (64.7%) IDU reported being hungry but not eating due to an inability to afford food.

Socio-demographic and drug-use characteristics associated with self-reported hunger in univariate analyses are shown in Table 1. Unadjusted factors associated with self-reported hunger among IDU included: being younger in age (Odds Ratio [OR] = 0.98, 95% Confidence Interval [CI]: 0.97-0.99, *p *= 0.005); downtown eastside residency (OR 2.07, 95% CI: 1.59-2.70, *p *< 0.001); living in unstable housing (OR 2.27, 95% CI: 1.74-2.96, *p *< 0.0001); spending ≥ CAD$50/day on drugs (OR: 2.00, 95% CI: 1.55-2.58, *p *< 0.001); and being incarcerated in the last six months (1.50, 95%CI: 1.06-2.11, *p *= 0.021). Symptoms of depression in the past week were also associated with self-reported hunger (OR 3.73, 95% CI: 2.80-4.98, *p *< 0.001). Drug use characteristics associated with self-reported hunger included: daily injection heroin in the last six months (OR 1.76, 95% CI: 1.32-2.34, *p *< 0.001); daily non-injection crack/rock in the last six months (OR 2.10, 95% CI: 1.60-2.74, *p *< 0.001); and any injection or non-injection drug binge in the past six months (OR 1.66, 95% CI: 1.28-2.14, *p *< 0.001).

**Table 1:** Factors associated with food insufficiency among injection drug users (n = 1,053)

Characteristics	Yes 65 (%) N = 681	No 35 (%) *N = 372*	***Odds Ratio*** (95% CI)	**Chi-Square**^**†, ‡**^	*p - *value
**Socio-demographic**					
**Age**					
Median (IQR)	41.1 (34.5-46.7)	42.8 (35.9-49.4)	0.98 (0.97, 0.99)	7.75	0.005
**Gender**					
Male	455 (66.81%)	245 (65.86%)	1.04 (0.80,1.36)	0.10	0.754
Female	226 (33.19%)	127 (34.12%)			
**Aboriginal identity**					
Yes	189 (27.75%)	118 (31.72%)	0.83 (0.63, 1.09)	1.83	0.176
No	492 (72.25%)	254 (68.28%)			
**DTES Residency**					
Yes	496 (72.83%)	210 (56.45%)	2.07 (1.59, 2.70)	29.22	< 0.001
No	185 (27.17%)	162 (43.55%)			
**Unstable housing**					
Yes	504 (74.01%)	207 (55.65%)	2.27 (1.74, 2.96)	36.99	< 0.0001
No	177 (25.99%)	165 (44.35%)			
**Education status**					
High school or greater	345 (50.66%)	205 (55.11)	0.84 (0.65, 1.08)	1.91	0.167
Other	336 (49.34%)	167 (44.89%)			
**Money spent on drugs per day**					
≥ $50	422 (61.97%)	167 (44.89%)	2.00 (1.55, 2.58)	28.46	< 0.001
< $50	259 (38.03%)	205 (55.11%)			
Incarceration **					
Yes	138 (20.26%)	54 (14.52%)	1.50 (1.06, 2.11)	5.33	0.021
No	543 (79.74%)	318 (85.48%)			
**Clinical**					
Symptoms of depression *					
**≥ 16 CES-D**	456 (66.96%)	159 (42.74%)	3.73 (2.80, 4.98)	88.19	< 0.001
**< 16 CES-D**	136 (19.97%)	177 (47.48%)			
**Drug Use**					
Daily injection heroin**					
Yes	240 (35.24%)	88 (23.66%)	1.76 (1.32, 2.34)	15.06	< 0.001
No	441 (64.76%)	284 (76.34%)			
Daily non-injection crack/rock **					
Yes	321 (47.14%)	111 (29.84%)	2.10 (1.60, 2.74)	29.75	< 0.001
No	360 (52.86%)	261 (70.16%)			
Daily injection cocaine**					
Yes	69 (10.13%)	28 (7.53%)	1.39 (0.88, 2.19)	1.95	0.162
No	612 (89.87%)	344 (92.47%)			
Any injection or non-injection crystal meth**					
Yes	28 (4.11%)	9 (2.42%)	1.73 (0.81, 3.71)	2.03	0.154
No	653 (95.89%)	363 (97.58%)			
Any injection or non-injection drug binge**					
Yes	352 (51.69%)	146 (39.25%)	1.66 (1.28, 2.14)	14.94	0.001
No	329 (48.31%)	226 (60.75%)			
**Daily alcohol use**					
< = 4 drinks	496 (72.83%)	279 (75.00%)	1.12 (0.84, 1.49)	0.58	0.446
> 4	185 (27.17%)	93 (25.00%)			
**Difficulty accessing drug/alcohol treatment**					
Yes	38 (5.58%)	14 (3.76%)	1.51 (0.81, 2.83)	1.69	0.193
No	643 (94.42%)	358 (96.24%)			

* Data missing for 125 participants

** In the past six months

^† ^All Chi-Square tests were performed with one degree of freedom (df = 1), with the exception of the

'symptoms of depression' variable, where df = 2.

‡ Chi-Squared Test were applied to all categorical variables. A Wald Test was applied to the continuous

'age' variable, yielding a Wald Chi-Square value.

Results from the multivariate analysis are shown in Table 2. Variables independently associated with self-reported hunger in multivariate analysis included: unstable housing (adjusted odds ratio [AOR]: 1.68, 95% confidence interval [CI]: 1.20 - 2.36, p = 0.003), spending > $50/day on drugs (AOR: 1.43, CI: 1.06 - 1.91, p = 0.018), and symptoms of depression (AOR: 3.32, 95% CI: 2.45 - 4.48, p = < 0.001). The statistical significance of these variables was found to be unchanged when re-estimating the multivariate model with removal of non-significant variables.

**Table 2:** Logistic Regression analysis of factors associated with food insufficiency among injection drug users (n = 1,053)

Variable	Adjusted Odds Ratio (AOR)	95% Confidence Interval (95% CI)	**Wald Chi-Square**^**†, ‡**^	p - value
**Age** (per year increase)	0.994	(0.98 - 1.01)	0.65	0.420
**DTES residency** (yes vs no)	1.34	(0.96 - 1.89)	2.89	0.089
**Unstable housing** (yes vs no)	1.68	(1.20 - 2.36)	9.04	0.003
**Money spent on drugs per day** (≥ $50 vs < $50)	1.43	(1.06 - 1.91)	5.64	0.018
Incarceration ** (yes vs no)	1.16	(0.79 - 1.69)	0.57	0.451
Symptoms of Depression* (≥ 16 CES-D vs < 16 CES-D)	3.32	(2.45 - 4.48)	62.79	< 0.001
Daily injection heroin ** (yes vs no)	1.27	(0.92 - 1.75)	2.05	0.152
Daily non-injection crack/rock ** (yes vs no)	1.18	(0.86 - 1.61)	1.02	0.313
Any injection or non-injection drug binge ** (yes vs no)	1.15	(0.86 - 1.53)	0.90	0.342

* Values for 125 participants missing from univariate treated as a separate category

** In the past six months

^† ^All Chi-Square tests were performed with one degree of freedom (df = 1), with the exception of the

'symptoms of depression' variable, where df = 2.

‡ Chi-Squared Test were applied to all categorical variables. A Wald Test was applied to the continuous

'age' variable, yielding a Wald Chi-Square value.

The p-value for Hosmer and Lemeshow Goodness-of-Fit Test was greater than 0.05, which indicates the model fit the data at an acceptable level. The concordance index was greater than 0.5, which implies a good probability of concordance between predicted and observed responses. The tolerance and variance inflation factor were examined for each variable, all of which were greater than 0.2 and less than 5.0, respectively, indicating no concerns with multicollinearity in the final model.

## Discussion

We found a very high prevalence of self-reported hunger among IDU in this Canadian setting, with 65% of participants meeting criteria for hunger. This is 62% higher than the prevalence severe food insecurity reported by the general Canadian population [20]. Self-reported hunger in this population was associated with low socio-economic status, symptoms of depression, and competing demands for food and drugs.

We found that self-reported hunger was not associated with use of specific types of illicit drugs. Rather it appeared to be predicted by the amount of drug use, as approximated by expenditures on drugs. Sixty two percent of hungry IDU spent over CAD$50/day, or > CAD$18,000/year. Similar habitual drug expenditures have been reported by cocaine and heroin users in the United States (U.S.) [21]. Our findings build upon previous studies that have found that IDU using multiple drugs, and over a long period of time, have significantly higher odds of being nutritionally deficient [4]. Drug addiction has shown to modify eating habits, often causing individual to eat fewer meals during a usual week [22], to skip meals for an entire day [23] and to prioritize drugs over everything else, including food intake [24]. Qualitative interviews with inner-city drug-using women reveal that the decision to buy drugs instead of food, even when hungry, is rationalized by the fact that food can be obtained for free from distribution sites, but getting a 'fix' always costs money [25]. Our findings suggest that IDU in this setting may benefit from improved access to drug treatment services to prevent deterioration of nutritional status.

We also found that 74% of IDU reporting hunger in our cohort were unstably housed. This proportion is elevated when compared to findings from a 2006 study within the same cohort, which found that 60% of IDU reported living in a single room occupancy hotel, shelter, recovery or transition house, jail, on the street or at no fixed address [16]. The association between hunger and unstable housing suggests that some IDU may not be accessing adequate nutritional services or housing needed to support food security. Over the past 20 years, a conflation of factors have contributed to high levels of homelessness in the Vancouver downtown eastside, where the majority of participants reside, including government deinstitutionalization of people suffering mental illness and addiction, rapid gentrification of the neighborhood, and insufficient low-cost housing options [17]. As part of the gentrification process, illicit drug users have also been the subject of ongoing police crackdowns on drug use that have forced them into peripheral urban areas [26]. In addition to our data showing that IDU are not accessing housing services [16], recent studies have found that IDU in Vancouver have limited access to essential harm reduction services [26] and delayed access to HIV clinical services [27]. Taken together, these data suggest the need to scale up multiple interventions to improve the health and wellbeing of IDU, and thereby reduce the problems of food insecurity and hunger among this population.

We found that 67% of IDU reporting hunger experienced symptoms of depression in the past week and that depression was strongly correlated with hunger. Cross-sectional research suggests that there is a strong association between household food insufficiency and adverse mental health. Food insufficiency has been associated with elevated incidence of major depressive disorders among women and adolescents in the general U.S. population [28,29]. Food insecurity has been associated with poor mental health status in HIV-positive populations, which include high proportions of illicit drug users [30-32]. No studies to our knowledge have assessed the correlation between self-reported hunger and symptoms of depression among IDU. Our findings suggest that this population would benefit from receiving mental health screening and support services as part of a comprehensive approach to improving their food security status and mental health.

There are several strengths and limitations to our study. Self-reported hunger is considered to be a severe manifestation of food insecurity [14]. Our measure of hunger has been validated in five North American settings, and has been associated with insufficient food intake [15]. The elevated prevalence of self-reported hunger among IDU in this study therefore likely indicates actual food inadequacy and severe food insecurity.

Because this is a cross-sectional study, we were unable to determine causality, or the biological and social pathways linking self-reported hunger and explanatory variables in this cohort. Longitudinal studies would be valuable to inform understanding of the mediating effect of socio-demographic and drug-use variables on food insufficiency. Longitudinal studies would additionally strengthen the power of similar analyses by enabling the inclusion of time-variant individual differences, and by helping to unpack the direction of causality between food insecurity and relevant demographic and clinical variables.

Common to all studies, it is possible that our study was affected by response bias. Studies evaluating nutritional status have found that self-reported nutritional measures are less reliable than clinical nutrition measures. Research has found that individuals tend to under-report energy intake, weight and BMI, and over-report height [33]. Self-reports of nutritional information often vary by gender and beliefs of social desirability [33].

Future evaluations of food insufficiency among IDU should seek to identify the unique social and biological factors mediating hunger. This is important in light of studies showing that a high proportion of drug users report disturbances in their social and familial networks, are underweight, and exhibit anorexia with poor food and drink consumption [5]. Further studies should consider exploring the impact of social support systems on subjective and objective measures of food insufficiency. A comprehensive nutritional evaluation, including dietary recall and body mass index (BMI) would also be valuable given the association between fasting appetite sensations with weight loss [34].

## Conclusion

We found a high prevalence of self-reported hunger among IDU in this marginalized urban setting. Hunger in this population is associated with poor socio-economic status, symptoms of depression and competing demands for food and illicit drugs. Our findings support the need for targeted social, mental health and nutritional support strategies for IDU, including addiction treatment programs that may reduce the adverse effect of ongoing drug use on hunger, and visa versa. Further studies should seek to identify behavioral and biological pathways leading to food insufficiency in this population, and evaluate the effectiveness of support services in mitigating this adverse outcome.

## Competing interests

The authors declare that they have no competing interests.

## Statement of Authors' contributions

All authors read and approved the final manuscript. AA, TK and SW designed research (project conception, development of overall research plant, and study oversight); JQ performed statistical analysis; AA, TK and SW wrote and made major contributions to the manuscript; JM and EW provided essential critical feedback on the manuscript; and TK had primary responsibility for final content.
